# Healing period after open high tibial osteotomy and related factors: Can we really say that it is long?

**DOI:** 10.1186/s40064-016-1745-0

**Published:** 2016-02-12

**Authors:** Masamichi Yokoyama, Yasuhiro Nakamura, Toru Onishi, Koji Hirano, Motoyuki Doi

**Affiliations:** Department Orthopedics, Okayama Kyokuto Hospital, Kurata 567-1, Naka-ku, Okayama City, Okayama 703-8265 Japan

**Keywords:** Degenerative gonarthrosis, Surgical management, High tibial osteotomy, Treatment period

## Abstract

High tibial osteotomy (HTO) is a general procedure for the treatment of degenerative gonarthrosis. In recent years, it has been reported that opening wedge high tibial osteotomy (OWHTO) has become widespread with good results. Despite these facts, HTOs tend to be avoided due to the need for long-term postoperative treatment. To investigate the treatment period for total recovery (healing period) after OWHTO and the factors affecting it. There were 47 cases of medial type degenerative gonarthrosis who underwent OWHTO from 2008 through 2011. The definition of the healing period was based on the time-dependent changes of the Japanese Orthopaedic Association score, focusing especially on pain on walking and pain on ascending/descending stairs. This score was defined as the Ability score. In this study, the healing period ended when the Ability score reached its maximum or when it showed a perfect score. Patients’ characteristics were examined to determine their effect on the healing period. The Ability score was 36.7 ± 10.1 (mean ± SD) before surgery and 51.6 ± 5.4 12 months after OWHTO. The healing period was 6.3 ± 3.9 months. Factors correlated with a longer healing period included female sex (correlation coefficient −0.35) and high BMI (correlation coefficient 0.33). Our study suggested that the healing period after OWHTO is approximately 6 months, and patients’ BMI and sex appear to be related to this period. This information is expected to be helpful for counseling patients undergoing treatment for gonarthrosis.

*Level of evidence* Therapeutic study, Level IV.

## Background

High tibial osteotomy (HTO) is a major surgical procedure that improves the condition of the knee joint. In recent years, opening wedge high tibial osteotomy (OWHTO) has also been increasingly used. However, the number of HTOs performed has not increased much, even though there are increasing numbers of patients with degenerative gonarthrosis. The HTO procedure is technically difficult to perform, therefore, highly experienced surgeons are needed for successful outcomes. Another reason is the long treatment period for total recovery after HTO. The duration of the treatment period has become an important issue, and this time period has an effect even on the choice of surgical method. However, the details of the process for obtaining good results in the short-term after HTO have not been well reported. Therefore, one must question whether there is any definitive evidence to suggest that healing takes a long time after HTO.

Thus, this study aimed to determine the actual period for total recovery (healing period) and to identify factors affecting the length of the healing period after the procedure.

## Patients and methods

A total of 47 cases (13 males, 34 females; mean age 59.8 years) who underwent OWHTO for medial type degenerative gonarthrosis that were unresponsive to conservative treatment between 2008 and 2011 were evaluated. In fact, OWHTO was carried out for 62 cases in total, but cases of simultaneous bilateral knee joint surgery and cases in which each patient’s femoro-tibial angle (FTA) was not corrected to within the general target angle of 167°–172° after the operation were excluded. FTA should be around 170° after OWHTO for better outcomes, and the surgery is planned to correct FTA to that degree. Therefore, cases with FTA outside of this range were excluded.

All patients had medial type degenerative gonarthrosis with grade 2, 3, or 4 of the Kellgren–Lawrence (K–L) classification (Table 1).

**Table 1:** Data of examined patients

Cases	47
Age (mean ± SD°(range))	59.8 ± 9.5 (35–73)
Sex	13 Males
	34 Females
K–L classification	18 Grade II
	28 Grade III
	1 Grade IV
BMI (kg/m^2^) (mean ± SD°(range))	26.2 ± 3.4 (20.6–37.0)

*SD* standard deviation, *K–L* Kellgren Lawrence, *BMI* body mass index

The following preoperative characteristics of the patients were evaluated: age; sex; K–L classification; body mass index (BMI); FTA measured on whole standing lower limb X-rays; mechanical axis percentage (%MA) where the Mikulicz line goes through the tibial plateau in the range of 0 in the internal edge to 100 in the external edge on the X-ray; posterior tibial slope (PTS); and JOA score. The FTA, %MA, and PTS were also evaluated postoperatively. The tibia was opened to the angle that was the same as the wedge-shaped spacer used in the operation. This was evaluated as the opening angle. Furthermore, for detailed evaluation, the JOA score was evaluated by physiotherapists 1.5 months after OWHTO and then every 3 months for 1 year.

**Fig. 1:**
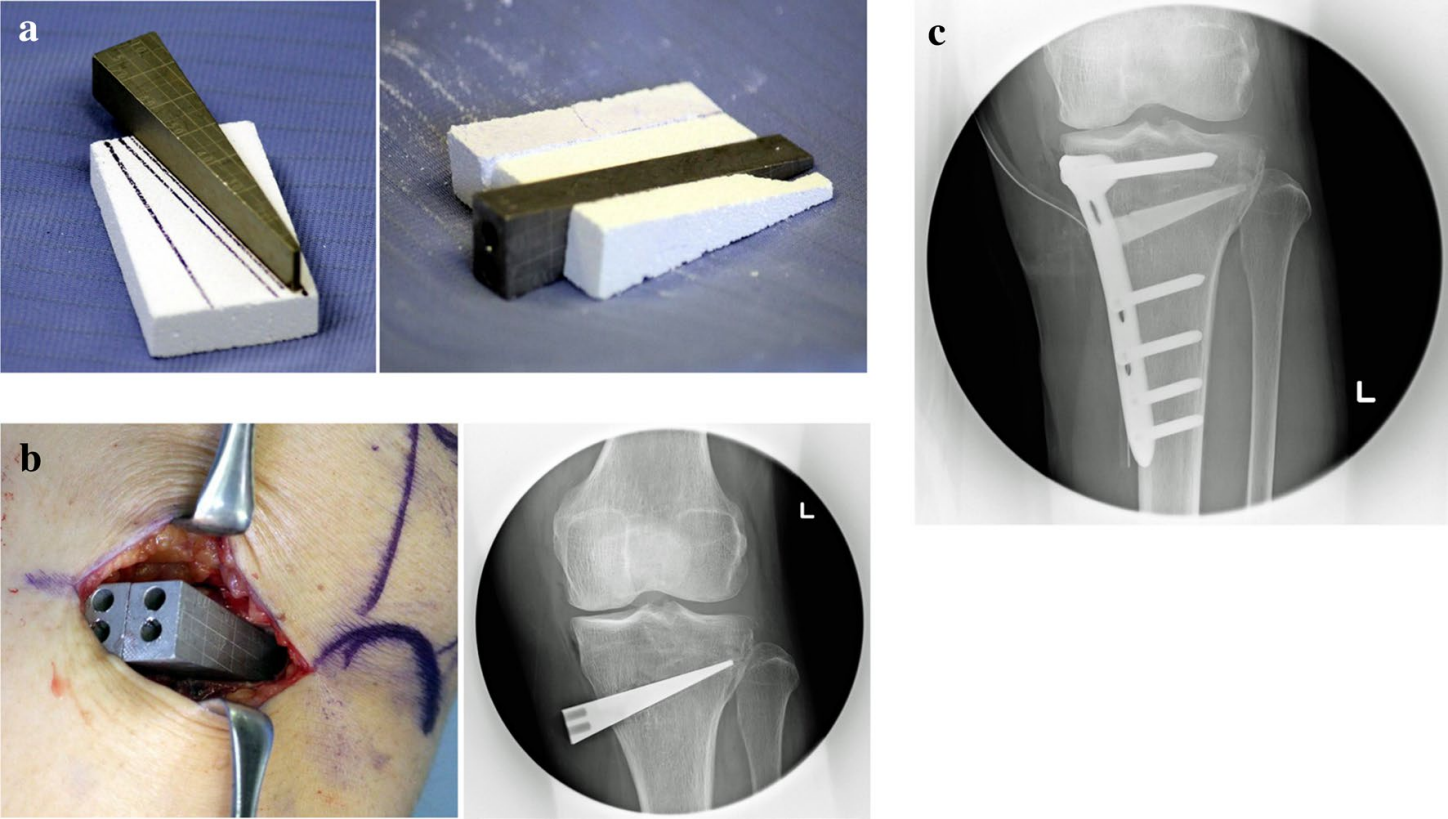
**a** A spacer is prepared to achieve the correction angle, and an OSferion is formed in the same shape as the spacer. **b** The cut tibia is opened with the spacer, and its condition is checked using fluoroscopy. **c** The spacer in the opening space is substituted by the same-shaped OSferion and is fixed with a TomoFix plate

The surgery was planned to correct the weight-bearing line of the lower limb to be about 63 %, the proper angle for the mechanical axis, called Fujisawa’s point. The tibia was opened with a wedged spacer set at this angle, and a United States Food and Drug Administration (FDA)-approved OSferion60 (β tricalcium phosphate, Olympus Terumo Biomaterial Corporation, Tokyo, Japan) of the same shape was placed inside. Then, internal fixation was completed with an FDA-approved Plating system (TOMOFIX system, DePuy Synthes Companies, Zuchwil, Switzerland) (Fig. 1). All cases were treated with this surgical technique by one surgeon.

**Fig. 2:**
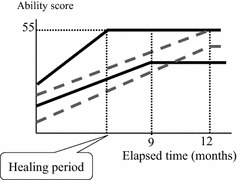
Patients in the Early Cured group heal within 9 months (shown as the *solid line*) with the defined end point, which shows a full score (55 points) or no further increase in the Ability score. The Late Cured group patients require 12 months to heal (shown as a *dotted line*)

Rehabilitation started on the day after OWHTO with range of motion (ROM) training. In addition, from the 2nd day after the surgery, patients started walking training with a load. This load was not restricted, but was chosen according to the degree of each patient’s pain. The degree of pain was observed by the change of the JOA score with time. It was mainly evaluated on the basis of two JOA score domains, pain on walking and pain on ascending/descending stairs. This was defined as the Ability score. Two types of end points were then set in order to determine the healing period: either the score reached the perfect score, or it showed no further improvement (Fig. 2). According to this definition, all cases were divided into two groups for comparison, the Early Cured group (fully improved within 9 months) and the Late Cured group (required 12 months for healing).

This study was carried out according to the ethical requirements of our hospital.

## Results

### Improvement and healing period

The average data (mean ± SD) of the 47 cases are reported. Comparing preoperative and 1-year postoperative values, FTA was corrected from 178 ± 2.4° to 168.9 ± 2.2°. The values of %MA and PTS also showed improvement, from 24.9 % ± 11.6 % to 66.7 % ± 9.7 % and from 10.3 ± 3.0° to 13.8 ± 3.3°, respectively. The total JOA score increased from 71.7 ± 12.2 to 93.1 ± 7.4, and the Ability score improved from 36.7 ± 10.1 to 51.6 ± 5.4 (Table 2). The average healing period was 6.3 months; 38 cases were in the Early Cured group, while 9 cases were in the Late Cured group (Table 3).

**Table 2:** Changes before and after OWHTO in lower limb alignment and clinical condition

	Before HTO (mean ± SD°(range))	1 year after HTO (mean ± SD°(range))
FTA	178.0 ± 2.4 (172–185)	168.9 ± 2.2 (165–174)
%MA	24.9 ± 11.6 (0–50)	66.7 ± 9.7 (47–88)
PTS	10.3 ± 3.0 (4–16)	13.8 ± 3.3 (8–21)
JOA score	71.7 ± 12.2 (45–95)	93.1 ± 7.4 (70–100)
Ability score	36.7 ± 10.1 (10–55)	51.6 ± 5.4 (35–55)

*HTO* high tibial osteotomy, *OWHTO* opening wedge HTO, *SD* standard deviation, *FTA* femorotibial angle, *MA* mechanical axis, *PTS* posterior tibial slope, *JOA* Japanese Orthopaedic Association

**Table 3:** Comparison of patients’ characteristics between the Early Cured group and the Late Cured group

	Early Cured group (mean ± SE°(range))	Late Cured group (mean ± SE°(range))
Cases	38	9
Age	59.4 ± 1.5 (35–73)	61.4 ± 3.4 (42–70)
Sex	12 males	1 male
	29 females	8 females
FTA (before surgery)	178.5 ± 0.4 (173–185)	177.3 ± 0.9 (172–181)
FTA (after surgery)	169.0 ± 0.4 (165–174)	168.6 ± 0.7 (166–173)
%MA (before surgery)	24.1 ± 1.9 (0–48)	26.7 ± 5.2 (0–40)
%MA (after surgery)	66.8 ± 1.6 (47–88)	66.2 ± 2.8 (52–77)
PTS (before surgery)	10.4 ± 0.5 (5–16)	10.1 ± 1.3 (4–15)
PTS (after surgery)	13.8 ± 0.5 (8–21)	13.7 ± 1.5 (8–19)
BMI	25.7 ± 0.5 (20.6–32.0)	28.3 ± 1.6 (23.0–37.0)
Ability score (before surgery)	37.8 ± 1.7 (10–55)	32.2 ± 2.2 (25–40)
Opening angle	10.3 ± 0.4 (5.4–14.7)	9.1 ± 0.6 (6.4–11.7)
Treatment period (months)	6.3 ± 3.9 SD (1.5–12)	

*FTA* femorotibial angle, *MA* mechanical axis, *PTS* posterior tibial slope, *BMI* body mass index, *SD* standard deviation, *SE* standard error

### Statistical analysis of the Ability score

**Fig. 3:**
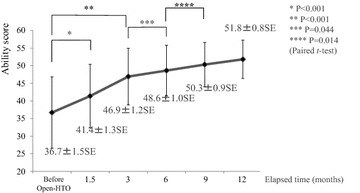
The score shows improvement at the 1.5-month evaluation, but there is no significant difference compared to before HTO. Three months after surgery, there is significant improvement from before HTO. The differences between the 3rd and the 6th month and between the 6th and 9th month are significant, even though there is only moderate improvement

Considering the changes of the Ability score over time, there was a trend to improvement at the 1.5-month evaluation (paired *t* test, P < 0.001), with a significant improvement at 3 months after surgery (paired *t* test, P < 0.001). There continued to be moderate but meaningful improvements at the 6- and 9-month evaluations (paired *t* test, P = 0.044 at the 6th month, P = 0.014 at the 9th month, Fig. 3).

### Comparing data of the two groups (Table 3)

**Fig. 4:**
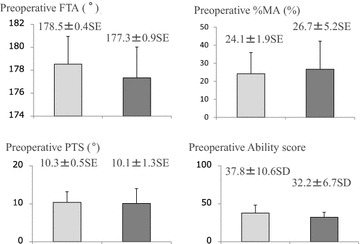
Comparing the patients’ preoperative characteristics, the BMI value is markedly higher in the Late Cured group than in the Early Cured group (Student’s *t* test P = 0.031). There are no other significant differences between the two groups

**Fig. 5:**
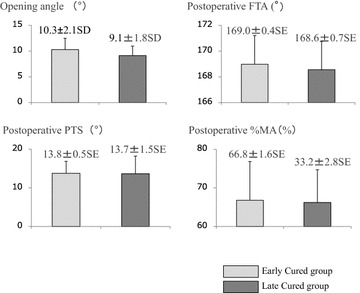
After the surgery, there are no significant differences in the correction angle, FTA, MA %, and PTS between the Early Cured group and the Late Cured group

Comparing the preoperative characteristics between the Early Cured group and the Late Cured group, the average BMI was significantly higher in the Late Cured group (28.3 ± 0.4 SE vs. 25.7 ± 1.6 SE; Student’s *t* test, P = 0.031, Fig. 4). The Ability score was also higher in the Early Cured group, but the difference was not significant. Other preoperative characteristics, including age, K–L classification, FTA, %MA, and PTS, showed no significant differences (Fig. 4). As for the postoperative examination, no differences were seen in the actual opening angle, FTA, %MA, and PTS (Fig. 5).

### Correlations

**Fig. 6:**
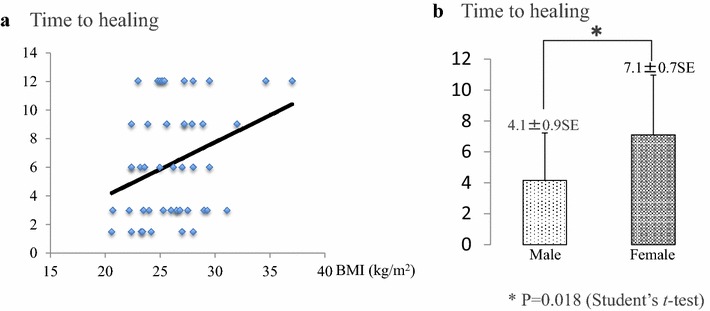
Factors affecting time to healing (using Pearson’s correlation coefficient). **a** Time to healing and BMI have a positive correlation (correlation coefficient 0.32). **b** Women take longer to heal than men (Student’s *t* test P = 0.018)

With respect to the correlation between the healing period and each factor examined, the healing period had a positive correlation with BMI (Pearson’s correlation coefficient: 0.32). In other words, a higher BMI value was associated with longer healing time (Fig. 6a). The healing period and patients’ sex were also correlated (Pearson’s correlation coefficient: 0.35). The healing period was significantly shorter in men (4.1 ± 0.9 SE months) than in women (7.1 ± 0.7 (SE) months; Student’s *t* test, P = 0.018) (Fig. 6b).

## Discussion

Surgeons seem reluctant to use HTO as a procedure due to the long healing period, although they make this decision primarily based on their own experience. In the present study, the healing period and the process involved after OWHTO were examined. Understanding these could be a strong motivation to recommend OWHTO over other procedures for the treatment of degenerative gonarthrosis.

During the treatment period after HTO, patients were evaluated using the JOA score. This scoring method consists of four items: pain on walking, pain going up and down stairs, range of motion, and hydrarthrosis. This study excluded the range of motion and hydrarthrosis scores from the evaluation so that the degree of recovery could be evaluated by the patient’s subjective symptoms. This score was evaluated over time, and the healing period was considered to have ended when the score stopped showing improvement after OWHTO. Since the condition of the knee joint after HTO is not shown on radiographic images, no imaging evaluation to observe the effect of treatment is available for this technique. Bone fusion of the osteotomy area was assessed by sequential CT images, and it was observed in all cases at the time of implant removal 1 year after surgery.

The results of the present study suggested an average 6-month healing period. In the follow-up examination after OWHTO, it is important to describe changes in the patient’s condition and the time required for relief of gonalgia. Although good postoperative short-term results are frequently reported, these results are based on improvement or recovery at the end of the examination (Jung et al. 2013; Yim et al. 2013). Takizawa et al. (1995) reported that their patients showed their first improvement 3 months after closed HTO. This improvement in the Ability score was obvious. At the 6-month evaluation, the score was much improved with the increase in muscular strength. In the present study, patients’ conditions seemed to be improved 1.5 months after OWHTO (Student’s *t* test, P < 0.001), but this decreased pain may have come from the low activity due to hospitalization. The difference became significant compared to the preoperative condition 3 months after surgery (Student’s *t* test, P < 0.001). The examination was carried out on all cases who were discharged from our hospital 3 months after HTO. Judging from these results, 3 months are required before improvement clearly starts, and the average healing period was 6.3 months, which is almost the same as that reported by Takizawa et al.

It is known that ROM, BMI, and other factors affect the long-term results after HTO. PTS, whose value usually increases after OWHTO (Noyes et al. 2005), is one of the major negative factors (Rodner et al. 2006). It has been reported that a high BMI can affect the treatment results. In the present study, a positive correlation was evident between BMI and the healing period. It follows that a high BMI value could also affect the healing period (Spahn et al. 2006). In addition, it has been found that less favorable results are achieved in women, though the reasons for this remain unclear (Niinimäki et al. 2012), and the present results showed the same tendency.

## Limitations

Patients were evaluated until the end of their healing period, up to a maximum of 12 months. This may represent a limitation of this study, as some patients included in the analysis from the Late Cured group were still experiencing pain 12 months after surgery.

It has been reported that some patients have pain caused by the TomoFix plate, but removal of the plate was not performed on any patient in the present study during the 12-month follow-up period. The plate in every case was removed after the 12-month period; it resulted in no apparent symptom improvement, and we think that the TomoFix plate had no effect on the present clinical results.

## Conclusion

In conclusion, this study suggested that the effect of OWHTO appeared in 3 months, and the average healing period was 6.3 months. Patients with a high BMI and women tended to have a longer healing period. These facts should be taken into account when choosing the appropriate surgical method and should be shared with patients with medial type degenerative gonarthrosis, because this can be important information for patients to decide whether to undergo OWHTO. Further studies on the process of treatment and related factors are needed to shorten the healing period.
